# AIMD - a validated, simplified framework of interventions to promote and integrate evidence into health practices, systems, and policies

**DOI:** 10.1186/s12874-017-0314-8

**Published:** 2017-03-04

**Authors:** Peter Bragge, Jeremy M. Grimshaw, Cynthia Lokker, Heather Colquhoun, The AIMD Writing/Working Group, Lauren Albrecht, Justine Baron, Ann Dadich, Laura Damschroder, Kristin Danko, Maria E. Fernandez, Signe Agnes Flottorp, Heather L. Gainforth, Kate Gooding, Ian D. Graham, Susanne Hempel, Simon Kitto, Jennifer Leeman, Danielle Mazza, Ann McKibbon, Susan Michie, Teryl Nuckols, John Ovretveit, Gjalt-Jorn Y. Peters, Hugo Sax, Shannon D. Scott, Kathleen R. Stevens, Michael G. Wilson

**Affiliations:** 1 BehaviourWorks Australia, Monash Sustainable Development Institute, Monash University, 8 Scenic Boulevard, Clayton, VIC 3800 Australia; 2 Clinical Epidemiology Program, Ottawa Hospital Research Institute and Department of Medicine, University of Ottawa, School of Epidemiology, Public Health and Preventive Medicine, 451 Smyth Road, Ottawa, ON K1H 8M5 Canada; 3 Department of Clinical Epidemiology and Biostatistics, McMaster University, 1280 Main Street West, Hamilton, ON L8S 4K1 Canada; 4 Department of Occupational Science and Occupational Therapy, University of Toronto, 160-500 University Ave, Toronto, ON M5G 1V7 Canada

**Keywords:** Knowledge translation, Implementation science, Framework validation, Healthcare quality improvement, Dissemination and implementation

## Abstract

**Background:**

Proliferation of terms describing the science of effectively promoting and supporting the use of research evidence in healthcare policy and practice has hampered understanding and development of the field. To address this, an international Terminology Working Group developed and published a simplified framework of interventions to promote and integrate evidence into health practices, systems, and policies. This paper presents results of validation work and a second international workgroup meeting, culminating in the updated AIMD framework [Aims, Ingredients, Mechanism, Delivery].

**Methods:**

Framework validity was evaluated against terminology schemas (*n* = 51); primary studies (*n* = 37); and reporting guidelines (*n* = 10). Framework components were independently categorized as fully represented, partly represented, or absent by two researchers. Opportunities to refine the framework were systematically recorded. A meeting of the expanded international Terminology Working Group updated the framework by reviewing and deliberating upon validation findings and refinement proposals.

**Results:**

There was variation in representativeness of the components across the three types of literature, in particular for the component *‘causal mechanisms’*. Analysis of primary studies revealed that representativeness of this concept lowered from 92 to 68% if only explicit, rather than explicit and non-explicit references to *causal mechanisms* were included.

All components were very well represented in reporting guidelines, however the level of description of these was lower than in other types of literature. Twelve opportunities were identified to improve the framework, 9 of which were operationalized at the meeting. The updated AIMD framework comprises four components: (1) **A**ims: what do you want your intervention to achieve and for whom? (2) **I**ngredients: what comprises the intervention? (3) **M**echanisms: how do you propose the intervention will work? and (4) **D**elivery: how will you deliver the intervention?

**Conclusions:**

The draft simplified framework was validated with reference to a wide range of relevant literature and improvements have enhanced useability. The AIMD framework could aid in the promotion of evidence into practice, remove barriers to understanding how interventions work, enhance communication of interventions and support knowledge synthesis. Future work needs to focus on developing and testing resources and educational initiatives to optimize use of the AIMD framework in collaboration with relevant end-user groups.

**Electronic supplementary material:**

The online version of this article (doi:10.1186/s12874-017-0314-8) contains supplementary material, which is available to authorized users.

## Background

Understanding and describing the science of effectively promoting and sustaining the use of research evidence in healthcare policy and practice is important to access literature; develop interventions; and plan, report, and review research. However, there are numerous terms to describe and conceptualise this science – for example Quality Improvement (QI), Implementation Science and Knowledge Translation. Compounding this, there are myriad theories and frameworks designed to guide the conceptualisation and development of interventions to promote evidence uptake into health practices, systems and policies [1–3]. Guidance on how to use these is lacking, evidence on the benefits of their use is sparse, and they themselves compound terminology issues by proposing an array of inconsistent terms for similar concepts. It is therefore unsurprising that theories and frameworks are only used to develop interventions about 10% of the time [4].

The existence of multiple terminologies and frameworks hampers advancement of this field [5, 6]. One of numerous examples is searching for literature; when McKibbon et al. developed search filters to identify literature on actions and processes of getting research findings used in practice, all of the filters had poor specificity (approximately 50–60%). This was because use of multiple terms resulted in large search yields containing many irrelevant articles [7].

To address this issue, an international collaboration of scholars – the Terminology Working Group - convened in September 2012 in Ottawa, Canada, to develop a simplified framework to describe interventions to promote and integrate evidence into health practices, systems, and policies. Whilst it appears counter-intuitive to address the problem of multiple frameworks with another framework, a key aim of the project was not to supplant existing models representing the full spectrum of activities in this field, but to create an overarching ‘meta-framework’ that accommodates the use of existing frameworks which are often designed for a more specific purpose. The framework was deliberately ‘terminology agnostic’ for two reasons – first, to prevent exacerbating the terminology issue; and second, to promote inter-sectoral collaboration across the diversely named but related fields that share the aim of improving healthcare quality. This ambition was reflected by the research group, which comprised members with diverse research foci including quality improvement, evidence synthesis, policy, information science, public health, patient safety and behaviour change. The group chose to focus on interventions because we considered that this contributes substantially to the terminology issue.

The resulting intervention framework comprised four components:

1. *Intended targets:* The intended effects of the intervention and/or its beneficiaries (e.g., behavioural changes, policy changes, technological changes);
2. *Active ingredients:* The critical components that define the intervention and are required to initiate change (e.g., educational workshop, opinion leader);
3. *Causal mechanisms:* The pathways or processes by which it is proposed that an intervention effects change or which change comes into effect. As with ingredients, other taxonomies could be used in conjunction with AIMD to add detail. The proposed mechanism could be based on either theory or empirical evidence (e.g. “the educational workshop is designed to address knowledge gaps and by providing new knowledge, influence practice”; “use of an opinion leader was successful in influencing the beliefs and attitudes of clinicians regarding the importance of hand hygiene”)
4. *Mode of delivery or application:* The ways in which active ingredients are applied (e.g., face-to-face, online, written material,).

This intervention framework, hereafter referred to as ‘simplified framework version 1,’ was published explicitly as a draft requiring further iteration [5]. This paper outlines results of a validation project and a subsequent second international meeting, and presents the updated intervention framework (simplified framework version 2), hereafter referred to as the AIMD framework [Aims, Ingredients, Mechanism, Delivery].

## Methods

### Validation project

Our operational definition of ‘framework validity’ was drawn from a study examining various methods in which multifaceted quality frameworks were validated:

“a process by which a judgement is made as to whether a tool is fit for purpose… the structure of a framework and the way in which it is validated should take into account the purpose for which the framework is to be used” (Inglis 2008 p. 350) [8].

This definition is distinguished from validity as (typically) applied to establishing the scope and the underlying constructs of measurement instruments [9]. The validation project addressed four research questions developed based upon the above definition:

1. *Does the simplified framework represent the domains described within existing terminology schemas?*

This validation question was designed to test whether our tool could be used as a common framework across diverse change literatures. simplified framework version 1 was validated against 51 terminology schemas identified in a systematic scoping review [10]. Examples of such schemas include the Consolidated Framework for Implementation Research (CFIR) [11] and the taxonomy of methods for implementing change in practice [12]. Two researchers [CL, JB or HC] independently adjudged representativeness of each component of the framework in each of the 51 terminology schemas.

2. *Is the simplified framework represented in published primary studies?*

This validation question aimed to evaluate whether relevant published research was already reporting the four domains identified in simplified framework version 1. Two researchers [LA, SDS] independently adjudged representativeness of the framework in 37 primary studies, sourced from four Cochrane [13–16] and six non-Cochrane systematic reviews [17–22] identified by the working group. These reviews were purposively sampled to encompass five sectors pertinent to promoting and supporting the use of research evidence in healthcare policy and practice: health behaviour change, patient safety, policy, public health, and quality improvement (two reviews per sector). Individual studies within each systematic review were also purposively sampled from to encompass different study designs and setting. The 37 identified primary studies pertained to health behaviour change (*n* = 8), patient safety (*n* = 5), policy (*n* = 8), public health (*n* = 8), and quality improvement (*n* = 8) and included 18 randomised controlled trials (RCTs), 5 before-after studies, 4 cohort studies, 4 interrupted time series studies, 3 cross-sectional studies, 2 controlled clinical trials, and an observational study.

Consideration of both explicit and non-explicit references to ‘*causal mechanisms’* was incorporated into analysis. An ‘explicit’ reference to a causal mechanism details how the intervention is proposed to alter healthcare practice, for example: “we postulated that provision of an education program would address identified gaps in knowledge amongst medical practitioners.” A ‘non-explicit’ reference describes the intervention, but the mechanism of action needs to be inferred by the reader, for example: “we provided an education program to medical practitioners.”

3. *Is the simplified framework represented in major reporting guidelines for interventions?*

This validation question aimed to evaluate whether relevant reporting guidelines recommended that intervention description encompass our four domains. We defined a relevant reporting guideline as one which was specific to reporting on complex interventions/interventions effectively promoting and sustaining the use of research evidence in healthcare policy and practice. All 280 reporting guidelines within the EQUATOR (Enhancing the QUAlity and Transparency Of health Research) Network online library (as at July 28, 2015) [23] were screened to identify relevant reporting guidelines. Two researchers [PB, AD] independently adjudged representativeness of the framework in the ten reporting guidelines identified following screening:

- Assessment of transferability and adaptation of health promotion interventions (ASTAIRE), [24];
- Consolidated Standards of Reporting Trials (CONSORT) Cluster Extension, [25];
- CONSORT Non Pharmacological Therapies (NPT) extension, [26];
- CONSORT Pragmatic Trials extension, [27];
- Criteria for Reporting the Development and Evaluation of Complex Interventions (CReDECI) 2, [28, 29];
- Reporting standards for studies of tailored interventions, [30];
- Template for intervention description and replication (TIDieR), [31];
- Transparent Reporting of Evaluations with Nonrandomized Designs (TREND), [32];
- Standards for QUality Improvement Reporting Excellence (SQUIRE) [33]; and
- Workgroup for Intervention Development and Evaluation Research (WIDER) [34].

For all three validation questions:

- Two researchers independently evaluated the representativeness of each component of the framework in the literature, where ‘fully’ represented was defined as the original framework component being represented in full; ‘partly’ was defined as the component being partially represented, but with some details unclear or missing; and ‘absent’ as no aspect of the component being referred to;
- Standardised data extraction forms were developed and piloted; and
- The weighted Cohen’s kappa statistic was used as a measure of categorical agreement between the two researchers [35]. Kappa calculations were performed in Microsoft Excel® using a linear weighting model where the weight of disagreement of Absent × Fully Represented was twice the weights of the other disagreements.

In examining framework representation across diverse research literature (questions 1–3), we postulated that more representativeness would equate to better framework validity, based upon the above definition.

Question four focused on how our validation work from questions 1–3 could refine simplified framework version 1:

4. *How can the useability of simplified framework version 1 be improved?* This validation question was designed to capture overarching information from the above three questions, for example whether there was any suggestion of missing domains or how clarity and/or language could be improved. Our intent was to use the answers to this question to guide framework refinements. We documented opportunities to potentially improve framework useability during the three validation studies. Opportunities to improve the framework were noted by members of the research team, either in separate word document files or in fields built into the data extraction form.

**Fig. 1:**
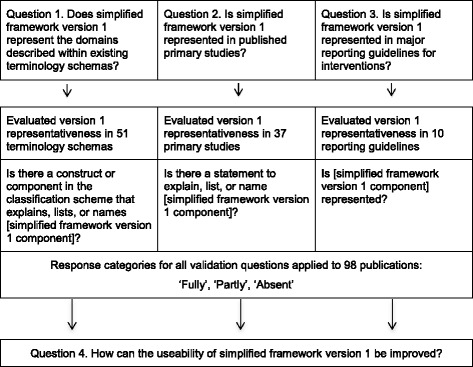
Summary of approaches to validation questions

Figure 1 summarises the methodological approach.

### 2nd International meeting

The Terminology Working Group that convened in September 2012 to develop simplified framework version 1 grew to 27 members as a result of the validation project. Twenty members of the group attended a 2-day working meeting in February 2015 in Ottawa, Canada. A full list of Terminology Working Group members involved in the project and their affiliations can be found in Additional file 1. The primary aim of this meeting was to refine the framework based upon the validation project. Meeting activities included a presentation on the background and the development of the original framework [5]; an overview of a scoping review conducted by members of the group on similar frameworks, i.e. classifications that describe interventions for the promotion of integration of evidence into practice, [10]; and presentation of results of the validation project, including potential refinements identified. A series of breakout and whole group discussions were facilitated by the meeting convenors (HC, JG, PB) to address two questions:

1. *Are the four existing framework components fundamentally important and critical?*
2. *How do we best present and define the four elements to optimize comprehension?*

## Results

### Validation project

Table 1 presents results pertaining to validation questions 1–3. Specifically, it presents the percentage of articles with each element represented across all included articles in each of the three types of literature for which framework validation was undertaken.

**Table 1:** Results from validation of the simplified framework by question (%)

Validation question [n publications]		Active ingredients	Causal mechanisms	Mode of delivery or application	Intended targets
Q1. Does simplified framework version 1 represent the domains described within existing terminology schemas?	Represented	96	59	73	80
	Fully	88	57	59	78
	Partly	8	2	14	2
	Absent	4	41	27	20
	Kappa	0.475: moderate strength of agreement^a^			
Q2. Is simplified framework version 1 represented in published primary studies?	Represented	81	92^b^	82	100
	Fully	70	92	59	100
	Partly	11	0	22	0
	Absent	19	8	19	0
	Kappa	0.725: substantial strength of agreement^b^			
Q3. Is simplified framework version 1 represented in major reporting guidelines?	Represented	95	100	95	95
	Fully	60	65	50	45
	Partly	35	35	45	50
	Absent	5	0	5	5
	Kappa	0.364: fair strength of agreement^a^			

^a^Interpretation of strength of agreement based upon Landis and Koch [35]

^b^Kappa based upon agreement when judgement of representativeness of causal mechanisms included both ‘explicit’ and ‘non-explicit causal mechanisms

*Question 1. Does simplified framework version 1 represent the domains described within existing terminology schemas?* At least one component of simplified framework version 1 was represented in 48 of the 51 terminology schemas. Representation rates ranged from 59% (causal mechanisms) to 96% (active ingredients). The range of full representation was 57–88% and the range of part representation was 2 to 14%.

*Question 2. Is simplified framework version 1 represented in published primary studies?*

At least one component of simplified framework version 1 was represented in all 37 primary studies. Representation rates ranged from 81% (active ingredients) to 100% (intended targets). The range of full representation was 60–100% and the range of part representation was 0 to 22%. In contrast to its representation in terminology schemas, *causal mechanisms* (92%) was highly represented in the primary studies. However, this included non-explicit causal mechanisms, identified with reference to the Behaviour Change Wheel [36]. A separate analysis based on only identifying explicit causal mechanisms resulted in a lower representation of *causal mechanisms* of 68% (comprising 41% full representation, 27% part representation and 32% non-representation).

*Question 3. Is simplified framework version 1 represented in major reporting guidelines?* At least one component of simplified framework version 1 was represented in all 10 reporting guidelines. Representation rates ranged from 95% (active ingredients, mode of delivery/application and intended targets) to 100% (causal mechanisms). The range of full representation was 45–65% and the range of part representation was 35 to 50%.

Weighted kappa scores for agreement between the two independent validation ratings represented a fair (0.364, reporting guidelines) to substantial (0.725, primary studies) strength of agreement.

*Question 4. How can the useability of simplified framework version 1 be improved?*

Twelve opportunities to refine the framework were identified, comprising four pertaining to the overall framework and two each pertaining to each of the four components. The four overall refinements gave important insights into the framework and how it could be used. For example, in the case of multifaceted interventions, while each facet may be considered an individual and equally important *active ingredient*, there may be various *causal mechanisms*, *modes of delivery*, and/or *targets* across the facets.

The eight opportunities pertaining specifically to the four components of version 1 revealed a need for conceptual clarity and more precise definition. For example, *active ingredient* appeared to overlap with *causal mechanisms* because knowledge of why an ingredient is *active* can be associated with knowledge of how an intervention is thought to work – for example, education could be considered both the *active ingredient* and *causal mechanism* in an education program. Relatedly, definitional issues were identified - for example, combining *mode of delivery* with *application* hindered the validation study, and furthermore, *mode of delivery* was thought to be only one of several factors that determine the replicability of an intervention.

### 2nd International meeting

Following deliberation across our diverse group, 9 of the 12 proposed refinements to simplified framework version 1 from question 4 were incorporated into AIMD; one of the four proposed overall framework refinements and all 8 opportunities pertaining specifically to the four components of version 1. In addition to considering the validation work conducted prior to the meeting, two other key concepts were also considered: 1) the original language for the descriptions of the framework components was difficult to apply, and 2) the framework had to remain congruent with other frameworks of implementation interventions for it to be applicable across disciplines and sectors. Table 2 presents a detailed description of refinements proposed through the validation and made in the meeting.

**Table 2:** Summary of evolution of simplified framework version 1 to the AIMD framework (simplified framework version 2)

Version 1 to updated version 2 components	Potential refinements identified	Refinements made: 2nd International meeting
Intended Targets to AIMS	Conceptually overlaps with causal mechanisms via reference to intended effects	Renamed as ‘Aims’ in version 2 and redefined to reflect that the intended effects are the aims of the intervention and the beneficiaries are who the aims are directed towards
	Evaluation could be considered part of intended target in that it quantifies the expected change; therefore intended target could be redefined to consider aim of study	See above
Active Ingredients to INGREDIENTS	Conceptually overlaps with causal mechanism (e.g., an active ingredient such as ‘persuasion’ implies a causal mechanism)	The word *active* was removed from version 1 to avoid confusion between the intervention ingredients and the mechanism by which the intervention works. This resulted in *‘Ingredients’* in version 2
	Best defined as ‘what it is’ (i.e., remove bulleted points, 3 and 4) or ‘as empirically established’	The single term *‘Ingredients’* acts as a prompt to provide details (i.e., the component parts) rather than a broad nominal description
Causal Mechanisms to MECHANISM	Conceptually overlaps with active ingredients as exemplified above	As above
	The term could refer to ‘how it is known to work’ (empirically established) or ‘how it is thought to work’ (theoretical rationale) – the definition could be refined or the concept of ‘rationale’ could be incorporated differently?	‘Causal’ was felt to indicate an empirically established mechanism, rather than a hypothesized mechanism. Therefore, ‘causal’ was removed from version 1 to allow for consideration of theoretical or empirical rationale. This resulted in *‘Mechanism’* in version 2
Mode of Delivery or Application to DELIVERY	Conceptually overlaps with active ingredient (e.g., local opinion leader implies active ingredient, i.e., a local opinion leader can deliver an intervention, but a local opinion leader is also an active ingredient, and could be delivered in multiple modes of delivery such as phone, face-to-face)	There can be more to delivery than just mode. Therefore, this component was renamed as *‘Delivery’* in version 2, and redefined to encompass information such as mode, content and dosage
	Mode of delivery alone is insufficient for replicability. Furthermore, combining mode of delivery with application is problematic as one may or may not be covered; this category may need to be redefined to include other vital information (e.g., eligibility criteria, mode, delivery personnel, content, dosage (i.e., duration, intensity), audience and its size of audience, number of care providers and centres, intervention fidelity and its measurement, the identification of breaches and how the intervention was modified, context, standardisation and tailoring strategies, clustering, blinding, enrolment, and allocation	Per above, *‘Delivery’* now defined more broadly to improve applicability to non-clinical settings (e.g., public health, policy)
Across all framework components	Consider how post-intervention information with potential application to future interventions could be used (e.g., fidelity, what was learned about causal mechanisms, contexts in which intervention may or may not be effective, financial considerations such as cost-benefit, etc.). This information could be considered as part of the description of a published study intervention or it could be assumed that this information is fed into future intervention studies (i.e., methods description)	The framework exists within the limits of the intervention itself; it does not extend to other contextual concepts (e.g. fidelity, rationale). Therefore, no refinements to version 1 were made pertaining to this issue
	Further guidance is needed on how to handle situations where there is more than one intervention (i.e., is the tool used per intervention or can a group of interventions be scored together?)	Multiple intervention components could be considered within AIMD. No refinements to version 1 were made pertaining to this issue
	‘Rationale’ for the intervention could be more explicitly covered, perhaps as a stand-alone component by the components and/or as a separate component	Not discussed at length in meeting. Changes were made to version 1 to more explicitly define ‘rationale’ within *‘Mechanism’* (see below)
	The control condition should be described in the same terms as the intervention and/or the control condition justified	Not discussed at length in meeting. No refinements to version 1 were made pertaining to this issue

### The AIMD framework

Table 3 presents the validated and revised version of the simplified framework version 1 - the AIMD framework.

**Table 3:** The AIMD Framework

Component	Description	Definition and considerations
Aims	What do you want your intervention to achieve and for whom?	This component relates to the objective and outcome of the intervention. Based on your endpoint, what are you measuring in whom? It could include consideration of proximal and intermediate outcomes, and process outcomes related to implementation.
Ingredients	What comprises the intervention?	These are the observable, replicable, and irreducible aspects of the intervention. To increase the detail specified, other taxonomies could be used in conjunction with the AIMD framework. This might include intervention taxonomies [38, 39] or reporting guidance [31].
Mechanism	How do you propose the intervention will work?	This refers to the pathways or processes by which it is proposed that an intervention effects change or which change comes into effect. As with ingredients, other taxonomies could be used in conjunction with AIMD to add detail. The proposed mechanism could be based on either theory or empirical evidence, and be made specific to the setting. The use of mechanism may change depending on if the framework is used for reporting or designing: why was the ingredient selected (design) and what is the pathway in which it worked (reporting).
Delivery	How will you deliver the intervention?	This encompasses logistical and practical information pertaining to intervention delivery, including mode (e.g. video, brochure); level (e.g. individual, team, population); dose, frequency, intensity; who’s delivering; and size of target group.

The AIMD framework retains the four components of its predecessor [5], yet includes simpler and clearer concepts and associated descriptions. For instance, ‘Intended Targets’ has been changed to ‘Aims’ to clarify that the component reflects what will be achieved and for whom; ‘Active’ has been removed from ‘Active ingredients’ to differentiate this component from the mechanism. Component descriptions were also simplified into short direct questions, as this was perceived in the meeting to be an easier way for end-users to engage with the concepts.

Given the pivotal importance of context in implementation science, there was considerable debate on whether (and how) to accommodate contextual factors that can shape the integration of research evidence into practice and policy. Recognizing their aim to address one aspect of implementation science, group members decided to: deem the framework as one element within the larger process of changing practice; maintain a focus on intervention characteristics, rather than the contextual factors that may or may not shape the intervention. Furthermore, although the importance of context is well-recognized [11], current understandings of this concept remain imperfect and incomplete. It was therefore decided to retain the four components in simplified framework version 1, rather than add a component pertaining to context. Future development of the AIMD framework could potentially elucidate the relationship between intervention and context.

## Discussion

Through a validation project and international meeting, we have updated a simplified framework of interventions to promote and integrate evidence into health practices, systems, and policies. Referred to as AIMD, the framework comprises four components: (1)Aims: what do you want your intervention to achieve and for whom?; (2)Ingredients: what comprises the intervention?; (3)Mechanisms: how do you propose the intervention will work?; and (4)Delivery: how will you deliver the intervention?

The validation process reported in this paper has several key strengths. First, it synthesised extensive input from the international research community with a broad range and large volume of literature, including literature sourced from systematic reviews. This is consistent with published framework validation strategies [8]. Second, in addition to evaluating the representativeness of the framework *in* relevant literature, it also involved gathering opportunities to improve the usefulness of the framework *from* the same literature. Third, all validation was independently completed by two independent researchers, with weighted kappa scores demonstrating a fair to substantial level of inter-rater agreement.

Despite these strengths, some methodological limitations warrant mention. First, the definitions of, ‘fully represented, ‘partly represented’, and ‘absent’ were established iteratively, rather than determined a priori. Notwithstanding this, the resulting definitions were similar across the three teams. Furthermore, through pilot testing and discussion, the definitions used to validate the framework were consistently applied within each team. Secondly, we acknowledge that the framework was evaluated predominantly by its developers. However, three of the 11 study authors (SS, DM, AD) were not involved in the initial framework development. Finally, it was not possible to map the framework to several reporting guidelines under development at time of study: the REporting Manualised INterventions for Dissemination and Evaluation (REMINDE) statement; CONSORT Extension for Social and Psychological Interventions: CONSORT-SPI; Consort extension to stepped wedge cluster randomised controlled trial; Guideline for reporting evidence based practice educational interventions and teaching (GREET) statement; Adapting TIDieR checklist for reporting public health, health systems and social and environmental policy interventions (UNTIDieR); Developing Standards for Reporting Phase IV Implementation studies (StaRI); and Reporting guidelines for implementation research and operational research [37]. As such, the strength of simplified framework version 1 with reference to these reporting guidelines is yet to be determined.

The representativeness of the simplified framework version 1 components varied across the three types of literature, reflecting the different questions being addressed through each of the validation exercises. Most notably, all components achieved at least 95% representation in the reporting guidelines. This is understandable given that guidelines are by nature a more comprehensive coverage of core concepts than primary studies. There was also considerable difference in the range of representation values in the reporting guidelines compared to the terminology schemas and primary studies.

The most substantial variation between the three validation studies at the level individual framework components was for *causal mechanisms,* which varied from 59% representation in terminology schemas to 100% in reporting guidelines. However, the validation findings for primary studies’ reporting of causal mechanisms (92% representation) should be interpreted in the context that non-explicit references to *causal mechanisms,* identified with reference to the Behaviour Change Wheel, [36] were included as ‘represented.’ According to a separate analysis, restricting the definition of ‘represented’ to explicit references to *causal mechanisms* substantially lowered the representativeness rate to 68%. This raises the question of how explicit reporting of causal mechanisms needs to be. One answer is that the detail required is that which maximises the replicability of a primary study. Non-explicit descriptions of causal mechanisms require these to be inferred by the reader, and this is therefore subject to individual reader knowledge and interpretation. For these reasons, we strongly advocate for explicit, rather than non-explicit descriptions of proposed causal mechanisms of interventions to promote uptake of evidence into policy. The much lower figure derived when limiting interpretation of reporting to explicit causal mechanisms indicates that this is an important area of future focus for the reporting of such interventions.

Overall, the variations in representativeness across the three types of literature indicate specific areas in which AIMD can contribute to addressing the terminology challenge. For example, the low representation of *causal mechanisms* (59%) in existing frameworks suggests that this aspect of intervention rationale is not well elucidated despite the existence of over 50 terminology schemas. The relatively low rate of ‘full’ representation of the components in reporting guidelines indicates that although the four components of the simplified model are present in this literature, there is scope to describe these with more granularity.

We anticipate several applications of the AIMD framework. AIMD can serve as a framework for more effectively communicating with each other about implementation interventions. Indeed, this benefit was realized at our meeting. Once we achieved consensus on terms and definitions, we were able to deploy these terms in rich discussions about interventions across multiple disciplines. This was because the group, having collaboratively developed and explicitly defined the four AIMD components, had a shared, unambiguous understanding of these elements of interventions. In explicitly defining its four components, the AIMD framework can also be used to guide the development of intervention design and reporting toolkits. If the term ‘AIMD’ and its associated elements were to become universally accepted terms and definitions for reporting, this could improve bibliographic searching for intervention studies over time by providing identifiers for more relevant citations and reducing the need for multiple synonyms, therefore reducing the ‘noise’ of irrelevant citations.

Potential users of this framework include patient safety agencies, clinicians, clinical quality improvement leads (who are not necessarily researchers), journal editors, implementation researchers, policy makers, patients, reporting guidance developers, funders, and those in public health. Dissemination practitioners and knowledge brokers could use the framework as a tool for planning, evaluating, and adapting knowledge dissemination materials. Future additional research on AIMD is required to establish how the AIMD framework can be used by the above stakeholder groups. Such research could address questions including:

- ‘Do the four components of AIMD inform and facilitate implementation, quality improvement, policy, patient safety?
- Does the framework promote comparisons between studies, elucidate issues of intervention fidelity, and further terminology within multi-faceted interventions?
- Can it stimulate knowledge discovery?
- To what extent can AIMD facilitate educational initiatives aimed at non-implementation audiences?’

A key future priority is to make AIMD useable to end-users, especially those involved in developing interventions to promote evidence-informed healthcare practice and policy. The true value of AIMD as a ‘meta-framework’ can only be realised when it is linked to existing, more specific intervention frameworks, worked examples and other resources that can aid intervention developers in addressing the four questions comprising the AIMD framework. The effectiveness of AIMD in optimising intervention design and reporting, as well as its impact on knowledge and application of theory, can then be evaluated.

## Conclusions

This study undertook a series of validation exercises of a simplified framework of interventions promoting and sustaining the use of research evidence in healthcare policy and practice. Using a definition of validity specific to frameworks – ‘whether a tool is fit for purpose’ – we validated the original framework’s four components (active ingredients, causal mechanisms, mode of delivery or application and intended targets) against terminology schemas, primary studies, and reporting guidelines. The results of the validation work, in addition to opportunities to improve the framework gathered through the validation activities, were used at an international and multi-sectoral meeting to refine the original simplified framework. The refined framework, AIMD, represents core components of implementation science interventions that are key to understanding an intervention, which will contribute to better understanding, design, evaluation, reporting, and communication in this field.

## Additional file

Additional file 1: Terminology Working Group members and affiliations. (DOCX 127 kb)

## Abbreviations

AIMD: The AIMD framework [Aims, Ingredients, Mechanism, Delivery]
ASTAIRE: Assessment of transferability and adaptation of health promotion interventions
CONSORT: Consolidated Standards of Reporting Trials
CReDECI: Criteria for Reporting the Development and Evaluation of Complex Interventions
EQUATOR: Enhancing the Quality and Transparency Of health Research
GREET: Guideline for reporting evidence based practice educational interventions and teaching
REMINDE: Reporting Manualised Interventions for Dissemination and Evaluation
SQUIRE: Standards for Quality Improvement Reporting Excellence
STaRI: Developing Standards forReporting Phase IV Implementation studies
TIDieR: Template for intervention description and replication
TREND: Transparent Reporting of Evaluations with Nonrandomized Designs
UNTIDIER: Adapting TIDieR checklist for reporting public health, health systems and social and environmental policy interventions
WIDER: Workgroup for Intervention Development and Evaluation Research
