# Supplementary material for: AIMD - a validated, simplified framework of interventions to promote and integrate evidence into health practices, systems, and policies
Source: BMC Med Res Methodol. 2017 Mar 4;17:38. doi: 10.1186/s12874-017-0314-8 (PMC5336675; doi:10.1186/s12874-017-0314-8)
Supplement: Additional file 1: — Terminology Working Group members and affiliations. (DOCX 127 kb) [file 12874_2017_314_MOESM1_ESM.docx]

**Additional File 1: Terminology Working Group members and affiliations**

| **Name** | **Affiliation** |
| --- | --- |
| Lauren Albrecht | Department of Pediatrics, Faculty of Nursing, University of Alberta, Canada |
| Justine Baron | Ottawa Hospital Research Institute, Ottawa, Canada |
| Peter Bragge | BehaviourWorks Australia, Monash Sustainable Development Institute, Monash University, Australia |
| Heather Colquhoun | Department of Occupational Science & Occupational Therapy, University of Toronto, Canada |
| Ann Dadich | School of Business, Western Sydney University, Australia |
| Kristin Danko | PhD student, Department of Epidemiology & Community Medicine,  University of Ottawa, Canada |
| Laura Damschroder | Ann Arbor VA Center for Clinical Management Research, HSR&D Center of Excellence, USA |
| Maria Fernandez | Professor of Health Promotion and Behavioral Sciences, University of Texas Health Science Center at Houston, School of Public Health, USA |
| Signe Agnes Flottorp | Senior Researcher, Department of Evidence Summaries,  Norwegian Knowledge Centre for the Health Services, Norway |
| Heather Gainforth | Centre for Outcomes Research and Effectiveness (CORE), Research Department of Clinical, Educational and Health Psychology, University College London, UK |
| Kate Gooding | Nuffield Centre for International Health and Development, University of Leeds, UK |
| Ian D Graham | Clinical Epidemiology Program, Ottawa Hospital Research Institute; School of Epidemiology, Public Health and Preventive Medicine, University of Ottawa, Canada |
| Jeremy Grimshaw | Canada Research Chair in Health Knowledge Transfer and Uptake, Ottawa Hospital Research Institute, Canada |
| Susanne Hempel | RAND Health, RAND Corporation, Santa Monica, United States |
| Simon Kitto | Department of Innovation in Medical Education,  University of Ottawa, Canada |
| Jennifer Leeman | School of Nursing, University of North Carolina, Chapel Hill, NC, United States |
| Cynthia Lokker | Health Information Research Unit, McMaster University, Canada |
| Danielle Mazza | Department of General Practice, Monash University, Clayton, Victoria, Australia |
| Ann McKibbon | Health Information Research Unit, McMaster University, Canada |
| Susan Michie | University College London, UK |
| Teryl Nuckols | RAND Corporation; Division of General Internal Medicine, Cedars-Sinai Medical Center, Los Angeles, USA |
| John Ovretviet | The Karolinska Institutet, Stockholm, Sweden |
| Gjalt-Jorn Peters | Open University, The Netherlands |
| Hugo Sax | Division of Infectious Diseases and Hospital Epidemiology, University Hospital of Zurich, University of Zurich; WHO Global Patient Safety Challenge Core Group; Switzerland |
| Shannon Scott | Canada Research Chair (Tier II) for Knowledge Translation in Child Health, AHFMR, Population Health Investigator, University of Alberta, Canada |
| Kathleen R Stevens | Academic Center for Evidence-Based Practice, University of Texas Health Science Center, San Antonio, USA |
| Michael Wilson | Department of Health Evidence and Impact, McMaster University, Canada |
